# First-Line Immune-Checkpoint Inhibitors in Non-Small Cell Lung Cancer: Current Landscape and Future Progress

**DOI:** 10.3389/fphar.2020.578091

**Published:** 2020-10-07

**Authors:** Zhangfeng Huang, Wenhao Su, Tong Lu, Yuanyong Wang, Yanting Dong, Yi Qin, Dahai Liu, Lili Sun, Wenjie Jiao

**Affiliations:** ^1^ Department of Thoracic Surgery, Affiliated Hospital of Qingdao University, Qingdao, China; ^2^ Department of Ultrasound, First Affiliated Hospital of Jinzhou Medical University, Jinzhou, China

**Keywords:** first-line, PD-L1, immunotherapy, checkpoints, non-small cell lung cancer

## Abstract

Lung cancer is one of the most common cancers and the leading cause of cancer-related deaths worldwide. Most of these patients with non-small cell lung cancer (NSCLC) present with the advanced stage of the disease at the time of diagnosis, and thus decrease the 5-year survival rate to about 5%. Immune checkpoint inhibitors (ICIs) can act on the inhibitory pathway of cancer immune response, thereby restoring and maintaining anti-tumor immunity. There are already ICIs targeting different pathways, including the programmed cell death 1 (PD-1), programmed cell death ligand 1 (PD-L1), and cytotoxic T lymphocyte antigen 4 (CTLA-4) pathway. Since March 2015, the US Food and Drug Administration (FDA) approved nivolumab (anti-PD-1 antibody) as the second-line option for treatment of patients with advanced squamous NSCLC. Additionally, a series of inhibitors related to PD-1/PD-L1 immune-checkpoints have helped in the immunotherapy of NSCLC patients, and modified the original treatment model. However, controversies remain regarding the use of ICIs in a subgroup with targeted oncogene mutations is a problem that we need to solve. On the other hand, there are continuous efforts to find biomarkers that effectively predict the response of ICIs to screen suitable populations. In this review, we have reviewed the history of the continuous developments in cancer immunotherapy, summarized the mechanism of action of the immune-checkpoint pathways. Finally, based on the results of the first-line recent trials, we propose a potential first-line immunotherapeutic strategy for the treatment of the patients with NSCLC.

## Introduction

The lung cancer is one of the most frequent malignant tumors, and ranks first in the incidence and mortality among all the cancer types globally (Bray et al., 2018). In clinical practice, only a small percentage of the patients with NSCLC are diagnosed at an early stage, while the majority of them present with locally advanced or metastatic disease at diagnosis, which accounts for their low five-year survival rate of 4–17% (Carbone et al., 2015; Hirsch et al., 2017; Osmani et al., 2018). Surgical resection remains the preferred treatment modality for the patients with early-stage NSCLC (Postmus et al., 2017). However, 58–73% of the patients with stage I and about 40% of those with stage II disease relapse after surgery, which reduces their 5-year survival rates (Liang and Wakelee, 2013; Gao et al., 2020). Whereas, different treatment methods are being adopted based on the overall health condition and the scope of the tumor in patients with NSCLC. Although platinum-based chemotherapy and radiation therapy are the traditional treatment methods for such tumors (Hanna et al., 2017; Tabchi et al., 2017), the last decade has shown the emergence of the molecular targeted therapies, including the tyrosine kinase inhibitors (TKI) targeting the epidermal growth factor receptor (EGFR), and the immune-checkpoint inhibitors (ICIs) that have helped improve the outcome of the patients with NSCLC (Hirsch et al., 2017; Dong et al., 2018).

A remarkable progress has been made in the field of molecular research in the last decade, which necessitated the World Health Organization (WHO) and the International Association for the Study of Lung Cancer (IASLC) to update the classification of lung cancer highlighting the molecular and immunohistochemical characteristics of the tumor subtypes (Travis et al., 2015; Osmani et al., 2018). The treatment of malignant tumors using immunotherapy has recently shown improvement (Kim and Chen, 2016). Moreover, immunotherapy has been approved since September 2014 for the treatment of metastatic melanoma (Robert et al., 2014), and the other tumor types, including the classical Hodgkin lymphoma (CHL) (Chen et al., 2017), renal cell carcinoma (RCC) (McDermott et al., 2016), head and neck squamous cell carcinoma (HNSCC) (Taylor et al., 2020), and NSCLC (Chen and Mellman, 2013; Ma et al., 2016; Queirolo and Spagnolo, 2017). Since March 2015, the nivolumab (anti-PD-1 monoclonal antibody) has been approved by the US Food and Drug Administration (FDA), as the second-line treatment for the patients with advanced NSCLC. Pembrolizumab (anti-PD-1 antibody), atezolizumab (anti-PD-L1 antibody), and durvalumab (anti-PD-L1 antibody) have been successively approved as the second-line, or even first-line, therapies for the patients with advanced NSCLC (Keating, 2015; Pai-Scherf et al., 2017; Antonia et al., 2018). However, despite the clinical benefits of immunotherapy, only a small proportion of the patients with NSCLC respond to ICIs administered as monotherapy, and not all responders continue to respond indefinitely (Kim and Chen, 2016). Most of patients treated with ICIs present with a variety of immune-related adverse events (IRAEs), including hepatitis, colitis, pneumonitis, thyroiditis, and so on. In addition, the severe adverse events can also have fatal consequences (Michot et al., 2016; Spain et al., 2016; Ali and Watson, 2017; Wang et al., 2017). Therefore, it is imperative to identify and analyses the NSCLC patients who can benefit from the treatment using ICIs.

Here, we have reviewed the history of the continuous developments in cancer immunotherapy. We present an analysis and summary of the completed and ongoing clinical trials with first-line immunotherapy and explore the possible models for their implementation for the treatment of patients with NSCLC.

## A Brief Overview of the Cancer Immunotherapy

**Figure 1:**
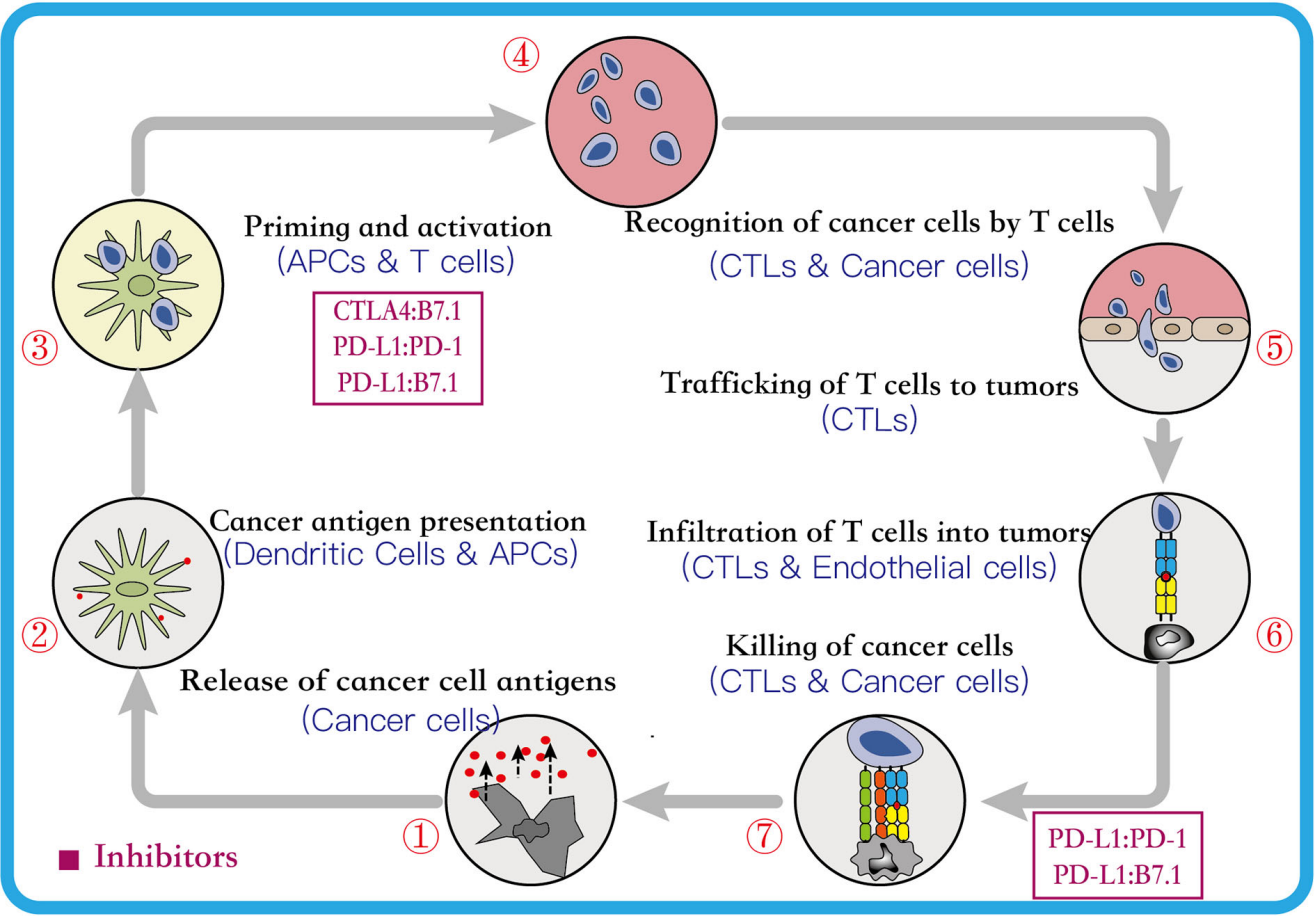
The Cancer-Immunity Cycle. This cycle can be divided into seven major steps, each major step is described above. Every major step in the cancer-immune cycle is regulated by stimulatory and inhibitory factors. The figure above lists two major inhibitory regulators. Immune-checkpoint proteins, such as CTLA4, can inhibit the development of an active immune response by acting primarily on T cell development and proliferation levels (step 3). Immunostat factors, such as PD-L1, can inhibit function that mainly acts to modulate active immune responses in the tumor bed (step 7) (Chen and Mellman, 2013). CTLA4, cytotoxic T lymphocyte antigen 4; PD-1, programmed cell-death 1; PD-L1, programmed cell-death ligand 1; APCs, antigen presenting cells; CTLs, cytotoxic T lymphocytes.

The cancer immunotherapy is a comprehensive concept that involves many aspects, including the human immune system, immunosurveillance, immune escape mechanisms, and the process of identifying and eliminating pathogens (Dermani et al., 2019). In cancer, studies have established that the immune system plays a dual role–it can either eliminate or suppress the cancer by inhibiting the growth of cancer cells, or enhance the growth of cancer by enriching cells that can evade the immunosurveillance or modify the tumor immune microenvironment suitable for the survival of cancer cells (Schreiber et al., 2011). Therefore, this dual-function of the immune system, as host-protective and tumor-promoting, is referred as cancer immune editing. It usually includes three consecutive phases, *viz.* elimination, equilibrium, and escape, and each phase is involved in the innate and adaptive immune responses (Vesely and Schreiber, 2013; Anagnostou and Brahmer, 2015). Further, the tumor cells can escape the immune system by decreasing the tumor antigenicity, reducing tumor immunogenicity and establishing an immunosuppressive tumor microenvironment (Beatty and Gladney, 2015; Jiang et al., 2019). Moreover, in the escape phase, the cancer cells recruit normal cells to establish an immunosuppressive tumor immune microenvironment, and eventually transform into malignant tumors (Hanahan and Weinberg, 2011). Though the administration of immunotherapy can promote the cytotoxic T lymphocytes to destroy the tumor cells, it requires a series of steps, called the Cancer-Immunity Cycle (CIC) (Chen and Mellman, 2013; Joyce and Fearon, 2015). The CIC usually includes seven primary continuous steps (**Figure 1**), which can be summarized as follows: (1) the cancer cells release neoantigens; (2) antigen-presenting cells (APCs) capture neoantigens released by the cancer cells; (3) APCs present the captured neoantigens to the T cells, which primes and activates the T cells; (4) the activated effector T cells are transported from the lymphoid organs to the tumor site *via* the circulatory system; (5) the effector T cells gradually infiltrate the tumor; (6) the activated effector T cells recognize the cancer cells in the tumors; and (7) the identified cancer cells are cleared by the effector T cells (Chen and Mellman, 2013; Karasaki et al., 2017). Thus, it can be concluded that the CD8^+^ T lymphocytes play an irreplaceable role, and each step can be regulated to either strengthen or weaken the CIC. Furthermore, studies suggest that the immune checkpoints can prevent T cell over-activation and maintain self-tolerance in the CIC. However, some tumor cells can recruit these checkpoint pathways to escape the immune system. Therefore, the administration of checkpoint inhibitors can block the association between the immune-checkpoint ligands and receptors in the CIC and prevent the immune escape, which maintains the function of the immune system and enhances the response of the effector T cells that eliminate the tumor cells (Pardoll, 2012; Anagnostou and Brahmer, 2015; Dermani et al., 2019). Additionally, several immune checkpoint combinations have been approved by the FDA for the treatment of the cancer patients with satisfactory results. The next section introduces their mechanism of action (Hargadon et al., 2018).

## Mechanism of Immune-Checkpoint Pathway in NSCLC

The introduction of the ICIs have been an important breakthrough in cancer treatment (Singh et al., 2020). The ICIs have proven their efficacy in the field of NSCLC and have been clinically approved by the FDA and/or the European Medicines Agency (EMA) (**Table 1**) (Xiao et al., 2020). The following sub-sections describe the mechanism of action of two immune checkpoints.

**Table 1:** Different immune-checkpoint inhibitors are approved for NSCLC.

Target	Drug	Trademark	Description	Manufacturer	FDA approval	Indication
PD-1	Nivolumab*	Opdivo	Fully human IgG4	Bristol-Myers Squibb	March 2015	Second-line treatment metastatic NSCLC
	Pembrolizumab*	Keytruda	Humanized IgG4	Merck	October 2015	First-line and second-line treatment NSCLC
PD-L1	Atezolizumab*	Tecentriq	Fully human IgG1	Roche	October 2016	Second-line treatment NSCLC
	Durvalumab	Imfinzi	Fully human IgG1	AstraZeneca	February 2018	Unresectable stage III NSCLC without relapse after chemo-radiotherapy
	Avelumab	Bevencio	Fully human IgG1	Merck Serono	–	Phase III
CTLA-4	Ipilimumab	Yervoy	Fully human IgG1	Bristol-Myers Squibb	–	Phase III
	Tremelimumab	–	Fully human IgG1	Astra Zeneca	–	Phase III

*Drug administration also approved by the European Medicines Agency (EMA); FDA, U.S. Food and Drug Administration; NSCLC, non-small cell lung cancer; PD-1, programmed cell death 1; PD-L1 programmed cell death ligand 1; CTLA-4: cytotoxic T lymphocyte antigen 4.

### Mechanism of Blocking the PD-1/PD-L1 Checkpoint Pathway

The PD-1 is a 288-residue type I transmembrane protein surface receptor. It is mainly expressed on the T lymphocytes in peripheral tissues and in small amounts on other immune cells, such as the dendritic cells (DCs), B lymphocytes, activated monocytes, natural killer (NK) cells, and myeloid-derived suppressor cells (MDSC) (Keir et al., 2008). The PDCD1, which encodes PD-1, was unexpectedly discovered by Professor Honjo and colleagues in 1992 while examining the mechanism of the programmed cell death pathway (Ishida et al., 1992). It consists of 5 exons located on the chromosome 2 in humans, and is homologous to the CD28 protein receptor family (Akinleye and Rasool, 2019). The PD-1 consists of extracellular, a transmembrane, and an intracellular domain. Further, the extracellular domain contains a single immunoglobulin V (IgV)-like domain, while the intracellular domain is made up of about 95-residues, and contains two phosphorylation sites that are the immunoreceptor tyrosine-based inhibitory motif (ITIM) and the immunoreceptor tyrosine-based switch motif (ITSM) (Nishimura et al., 1999; Keir et al., 2008; Zak et al., 2017). Additionally, the preliminary analysis have suggested that ITIM and ITSM, upon phosphorylation, bind to the protein tyrosine phosphatases (PTPs), such as SHP2, which negatively regulates the effector T cells (Topalian et al., 2015).

The PD-L1 (CD274) and PD-L2 (CD273) are known ligands of the PD-1 (He et al., 2004). The PD-L1 is a type I transmembrane protein consisting of 290 residues, and is encoded by the CD274 gene that contains 7 exons. Further, the CD274 is situated on the chromosome 19 in mice and on human chromosome 9 (Zak et al., 2017). The PD-L1 protein consists of three domains, *viz.* the transmembrane, intracellular, and extracellular that contains the IgV-like domain, IgC-like domain, and signal sequences (Keir et al., 2008). Furthermore, PD-L1 is expressed in different cell types, including the immune cells (APCs), non-lymphoid organs (the lung, heart, and placenta), and non-hematopoietic cells (epithelial cells, endothelial cells, and tumor cells), as opposed to the PD-1 that is primarily expressed in the immune cells (T- and B-lymphocytes) (Zhang et al., 2019). Whereas, the PD-L2 shows a limited expression range, detected in the B lymphocytes, macrophages, dendritic cells, and bone marrow-derived mast cells (Chen et al., 2016). Studies have shown that the expression of PD-L1 can be induced or regulated by a variety of pro-inflammatory cytokines in several cell types, and this effect is particularly prominent in the tumor cells (Akinleye and Rasool, 2019). Moreover, numerous inflammatory cytokines, including the IFN-γ, TNF-α, and IL-10 are secreted by the activated T cells and NK cells, of which IFN-γ shows a predominant effect (Li et al., 2018).

**Figure 2:**
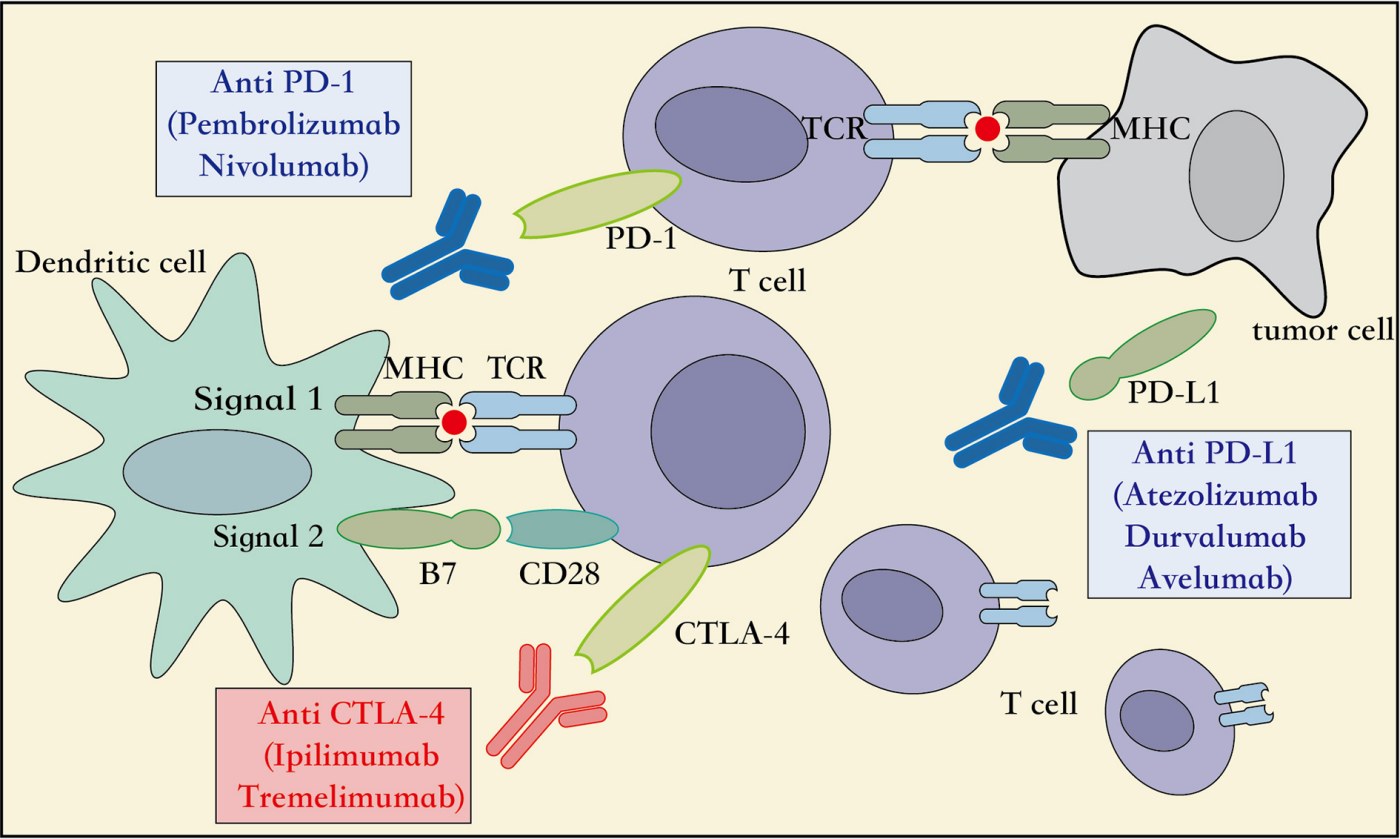
Mechanism of action of immune-checkpoint inhibitors. APC, Antigen presenting cell; CD28, cluster of differentiation 28; MHC, major histocompatibility complex; PD-1, programmed cell death 1; PD-L1, programmed cell death ligand 1; CTLA4, cytotoxic T lymphocyte associated protein 4, TCR, T cell receptor.

Several studies have showed that targeting the expression of PD-1 on the T lymphocytes and PD-L1 on the cancer cells can inhibit the function or cause dysfunction of the T lymphocytes, induce apoptosis of T lymphocytes, and promote the production of the cytokine interleukin 10 (IL-10) in the tumor microenvironment (Sun et al., 2015). Therefore, the tumor cells over-expressing PD-L1 can escape immune responses mediated by the cytotoxic T lymphocytes (CD8^+^) (Zou and Chen, 2008). Moreover, the other T cell subtypes, such as the regulatory T cells (Treg), create a highly immunosuppressive tumor microenvironment by maintaining PD-1 expression on their surface that further suppresses the effector immune response (Francisco et al., 2010). Thus, based on this mechanism of action, the PD-L1/PD-L2 expressed on the surface of tumor cells can be inhibited from binding the PD-1 expressed on the surface of the T lymphocytes, so that they can activate the innate or adaptive immune responses and destroy the tumor cells (**Figure 2**). Furthermore, the PD-1 and PD-L1 provide immune targets for immunotherapy and allow durable response in NSCLC.

### Mechanism of Blocking the CTLA4/CD28 Checkpoint Pathway

The CTLA-4, also known as CD152, is a receptor protein widely expressed on the surface of the T lymphocytes, B lymphocytes, and fibroblasts (Pardoll, 2012). In the third step of the CIC, the early stage of neoantigen presentation, the receptor CTLA4 on the surface of T lymphocytes competes with the co-stimulatory receptor CD28 to bind to the ligands B7-1 (CD80) and B7-2 (CD86) expressed on the APCs. However, with a higher affinity and lower surface density to bind the B7 ligand than the CD28 receptor, the CTLA4 receptor inhibits the association of CD28 to the B7 ligand. This reduces the production of the cytokine IL-2, which suppresses the immune response and prevents functioning of the CIC (Qureshi et al., 2011) (**Figure 2**). Therefore, an inhibitor of the CTLA4 checkpoint can suppress the association of the CTLA4 receptor to its ligand B7, and thus aid the immune cells to clear tumors through the activation of innate and adaptive immune responses. Moreover, immune-checkpoint inhibitors targeting the CTLA4, including the ipilimumab and tremelimumab, have been adopted by the US FDA as immunotherapy options for the patients with metastatic melanoma. Additionally, multiple randomized clinical trials using checkpoint inhibitors related to CTLA4, administered alone or in combination with other treatment modalities for NSCLC, are ongoing, and are expected to achieve better survival outcomes with acceptable toxicity levels (Hodi et al., 2010; Hellmann et al., 2018). Taken together, additional immunotherapeutic strategies targeting the CTLA4 checkpoints can be explored in several cancer types.

## First-Line Immune-Checkpoint Inhibitors Monotherapy for NSCLC

Several randomized controlled trials have confirmed that the patients treated with ICIs show better clinical outcomes than the patients receiving second-line docetaxel for the treatment of advanced NSCLC. This article lists the results of multiple clinical trials in **Table 2**, receiving monoclonal antibody monotherapy (including nivolumab, pembrolizumab, and atezolizumab) and platinum-based chemotherapy.

**Table 2:** Summary of immune-checkpoint inhibitors monotherapy as first-line treatment for advanced NSCLC.

Study	Phase	Sample size	Histology	RR, %	Median PFS (months)	Median OS (months)	Grade 3–5 TRAEs, %	Treatment arms	PD-L1 expression	Ref.
CheckMate-026 (NCT02041533)	III	423	Squamous and non-squamous	26 vs. 33	4.2 vs. 5.9	14.4 vs. 13.2	18 vs. 51	Nivolumab vs. chemotherapy	PD-L1 ⩾ 5%	(Carbone et al., 2017)
KEYNOTE-001 (NCT01295827)	Ib	550	Squamous and non-squamous	Treatment naïve 41.6		Treatment-naïve PD-L1 ⩾ 50% 35.4 PD-L1 1–49% 19.5	13	Pembrolizumab	PD-L1 unselected	(Garon et al., 2019)
				Previously treated 22.9		Previously treated PD-L1 ⩾ 50% 15.4 PD-L1 1–49% 8.5 PD-L1 < 1% 8.6				
KEYNOTE-024 (NCT02142738)	III	305	Squamous and non-squamous	44.8 vs. 27.8	10.3 vs. 6.0	30.0 vs. 14.2	26.6 vs. 53.3	Pembrolizumab vs. chemotherapy	PD-L1 ⩾ 50%	(Reck et al., 2016)
KEYNOTE-042 (NCT02220894)	III	1274	Squamous and non-squamous	PD- L1⩾50% 39 vs. 32	PD-L1 ⩾ 50% 7.1 vs. 6.4	PD-L1 ⩾ 50% 20.0 vs. 12.2	18 vs. 41	Pembrolizumab vs. chemotherapy	PD-L1 ⩾1%	(Mok et al., 2019a)
				PD- L1⩾20% 33 vs. 29	PD-L1 ⩾ 20% 6.2 vs. 6.6	PD-L1⩾;20% 17.7 vs. 13.0				
				PD-L1 ⩾ 1% 27 vs. 27	PD-L1 ⩾ 1% 5.4 vs. 6.5	PD-L1 ⩾ 1% 16.7 vs. 12.1				
BIRCH (NCT02031458)	II	Cohort 1 first line:139	Squamous and non-squamous	22	5.4	23.5	9	Atezolizumab	PD-L1⩾5%	(Peters et al., 2017)
FIR	II	Cohort 1 first line: 31	Squamous and non-squamous	32	5.5	14.4	16	Atezolizumab	PD-L1⩾5%	(Spigel et al., 2018)

OS, overall survival; PD-L1, programmed cell-death ligand 1; PFS, progression-free survival; RR, response rate.

### Nivolumab (ONO-4538/BMS-936558)

The nivolumab, targeting PD-1, is a humanized immunoglobulin G4 (IgG4) monoclonal antibody (Gettinger et al., 2015). The CheckMate-026 phase III clinical trial was initiated by our group to test the efficacy of nivolumab monoclonal antibody administered as a monotherapy. The patients (n = 541) with stage IV or recurrent NSCLC harboring PD-L1 positive tumors (PD-L1 ≥ 1%) were randomly recruited and grouped in a 1:1 ratio to either receive nivolumab or platinum-based chemotherapy. Of these, 423 patients showed PD-L1 expression more than 5% (PD-L1 ≥ 5%), and the progression-free survival (PFS) was used as the primary endpoint to assess the outcome. Our analysis indicated the nivolumab monotherapy to be ineffective in extending the PFS and overall survival (OS) than that achieved with chemotherapy in the control arm (PFS: 4.2 months versus 5.9 months; OS: 14.4 months versus 13.2 months; overall response rate (ORR): 26.1 versus 33.5%, respectively). Further, the nivolumab monotherapy showed significantly lower treatment-related adverse events (TRAEs) than treatment with chemotherapy (71 versus 92%), especially in the incidence of grade 3 or higher adverse events (17.6 versus 50.6%). However, in the nivolumab arm, though OS of patients with high tumor mutation burden (TMB) was low, the ORR and PFS were found to be significantly improved than those in the chemotherapy arm. Therefore, this analysis demonstrated the predictive value of TMB for evaluating the efficacy of immunotherapy in phase-III clinical trial (Carbone et al., 2017; Zarogoulidis et al., 2018).

### Pembrolizumab (MK-3475)

The KEYNOTE-001 trial was a phase Ib clinical study of pembrolizumab monotherapy administered in previously treated or untreated patients with advanced or metastatic NSCLC (Garon et al., 2015; Hui et al., 2017). The trial recruited a total of 550 patients with NSCLC, which included 101 untreated and 449 previously treated patients, and had a follow-up duration of more than 5 years. The data discussed at the American Society of Clinical Oncology (ASCO) meeting in 2019 showed 15.5% 5-year OS rate in patients in the previously-treated category. Of these patients, those in the PD-L1-high expression (PD-L1 ≥ 50%) arm showed 25.0% 5-year OS rate. Whereas, patients in the PD-L1 low (PD-L1: 1–49%) and PD-L1-negative (PD-L1 < 1%) arm showed 12.6 and 3.5% 5-year OS rate, respectively. However, in the previously-untreated category, patients showed 23.2% 5-year OS rate, which was found to be better in the patients in the PD-L1-high arm than those in the PD-L1-low arm (29.6 versus 15.7%) (Garon et al., 2019). Therefore, it can be concluded that, especially in the arm with high expression of PD-L1, treatment with pembrolizumab monotherapy could effectively prolong the survival outcome.

In a subsequent randomized phase III trial, KEYNOTE-024, 305 previously-untreated patients with advanced NSCLC having PD-L1 expression in more than 50% tumor cells and no *EGFR*/*ALK* mutations were recruited to either receive pembrolizumab or platinum-based chemotherapy (Reck et al., 2016). The patients treated with pembrolizumab showed better PFS, OS, and ORR than those treated with chemotherapy (Reck et al., 2016; Reck et al., 2019a). Moreover, the TRAEs were found to be 26.6 versus 53.3%, respectively, thus indicating the pembrolizumab monotherapy to be safe and better than chemotherapy. Based on these results, the US FDA approved the pembrolizumab as a single-agent first-line immunotherapy in patients with advanced NSCLC harboring high PD-L1 expression (PD-L1 ≥ 50%) and no *EGFR*/*ALK* mutations (Pai-Scherf et al., 2017). This approval by the FDA changed the landscape of first-line immunotherapy, and provided more treatment options for the patients with advanced NSCLC. In 2019, the 3-year survival follow-up data of the trial (KEYNOTE-024) was presented at the World Conference on Lung Cancer (WCLC). The results indicated that the pembrolizumab monotherapy significantly prolonged the median OS length (26.3 months versus 14.2 months), and the 3-year OS rate (43.7 versus 24.9%) than is those treated with standard chemotherapy. Additionally, the overall safety of immunotherapy was found to be better than chemotherapy (Reck et al., 2019b).

Further, the KEYNOTE-042 phase III clinical trial was set to benefit more patients from the pembrolizumab monotherapy and expand the beneficiary population. In this study, 1274 patients with advanced NSCLC who were previously-untreated and harbored tumors positive for PD-L1- expression and no *EGFR*/*ALK* mutations (Mok et al., 2019a; Mok et al., 2019b). All the patients were randomly divided into two arms to either receive pembrolizumab or chemotherapy. Each treatment arm was further divided into three subgroups based on the level of PD-L1 expression (PD-L1 ≥ 50%, ≥ 20%, ≥ 1%). In addition to comparing these three different expression levels, the fourth PD-L1 TPS is 1–49% as an exploratory endpoint. We defined the OS was used as the main endpoint of the trial. With a median follow-up duration of 12.8 months the updated data suggested that the patients treated with pembrolizumab monotherapy showed significantly better OS than those treated with chemotherapy (PD-L1 ≥ 50%: 20.0 months versus 12.2 months; PD-L1 ≥ 20%: 17.7 months versus 13.0 months; PD-L1 ≥ 1%: 16.7 months versus 12.1 months). Moreover, the OS of patients who received pembrolizumab monotherapy in the high PD-L1 expression arm (PD-L1 ≥ 50%) was prolonged by 7.8 months, and showed maximum benefit. At the same time, when we followed up to 24 months, the researchers compared the survival percentages of patients receiving pembrolizumab and chemotherapy and found that regardless of the level of PD-LI expression, the survival benefit was more obvious in the subgroup of patients receiving immunotherapy (PD-L1 ≥ 50%: 45 versus 30%; PD-L1 ≥ 20%: 41 versus 30%; PD-L1 ≥ 1%: 39 versus 28%). However, by comparing the PD-L1 low expression (PD-L1, 1–49%) and PD-L1 high expression (PD-L1 > 50%) arms, it is particularly important to note that pembrolizumab and platinum-based chemotherapy have similar median OS in the PD-L1 low expression group, which is no significant statistical difference [13.4 months versus 12.1 months; HR 0.92 (95% CI, 0.77–1.11)]. The reason for this phenomenon may be that the data in the PD-L1 TPS ≥ 1% and TPS ≥ 20% arms overlap with the data in the PD-L1 TPS ≥ 50% arm (Jørgensen, 2020). Thus, these results validate the benefits observed using the pembrolizumab in the KEYNOTE-024 trial, and supports its administration as a single-drug in patients with advanced NSCLC harboring PD-L1 ≥ 50%. Additionally, the incidence of grade 3 or severe TRAEs was 17.8 and 41% in the immunotherapy and chemotherapy arm, respectively, indicating lower propensity of adverse events upon pembrolizumab treatment (Mok et al., 2019a). Although, patients with high PD-L1 expression were notably exceeding those in the other subgroups of this trial. The analysis of the various subgroups indicated statistically insignificant difference in OS in patients with PD-L1 expression in the 1–49% range. Therefore, the trial KEYNOTE-042 failed to rewrite the guidelines issued by the FDA to confer benefit to the NSCLC patients.

Reviewing the above several clinical trial studies (CheckMate-026, KEYNOTE-024 and KEYNOTE-042), it was found that the first-line single-agent immunotherapy showed different results. In NSCLC patients with PD-L1 ≥ 5%, the use of nivolumab monotherapy to treat OS was not benefit compared to standard chemotherapy. In contrast, OS can significantly benefit from the use of pembrolizumab to treat PD-L1 ≥ 50% of patients with NSCLC. Therefore, it is suggested that patients should be highly selected for first-line immune monotherapy in clinical practice. Therefore, in clinical practice, it is suggested that first-line immune monotherapy requires a predictive biomarker (such as PD-L1, etc.) to highly select patients who can produce sustained immune response. In 2019, R. de Vries and his colleagues found that exhaled breath analysis by electronic nose can screen out responders and non-responders to anti-PD-1 therapy in order to find patients who could benefit from immune monotherapy. In addition, the prediction effect of this new biomarker is significantly better than that of PD-L1, which is currently used clinically (de Vries et al., 2019).

### Atezolizumab (MPDL-3280A)

A phase II trial with atezolizumab, BIRCH, recruited patients with advanced NSCLC harboring PD-L1 ≥ 5% and no disease of the central nervous system. We set the objective response rate as the primary endpoint, and progression-free survival (PFS), median duration of response, and overall survival (OS) are secondary endpoints. The analysis suggested that advanced NSCLC patients treated with atezolizumab monotherapy showed better outcome in the primary endpoint (Peters et al., 2017), and the FIR results of this trial indicated possibility of clinical benefits that need further analysis (Spigel et al., 2018).

## First-Line Immune-Checkpoint Inhibitors Therapy Combined With NSCLC

The previous studies implicate treatment with chemotherapy to influence the immune response and enhance the expression of PD-L1. Data suggests that treatment with immunotherapy and chemotherapy may show a synergized effect. Therefore, several randomized clinical trials combining immunotherapy and chemotherapy are ongoing, and identification of a reasonable combination of these agents that can confer better survival outcome in patients with advanced NSCLC can be anticipated. The data of the ongoing clinical trials have been summarized in **Table 3**.

**Table 3:** Summary of immune-checkpoint inhibitors combined with other therapies advanced NSCLC.

Study	Phase	Sample size	Histology	RR, %	Median PFS (months)	Median OS (months)	Grade 3–5 TRAEs, %	Treatment arms	PD-L1 expression	Ref.
Combination immune-checkpoint inhibitor and chemotherapy										
KEYNOTE-021(NCT02039674)	II	123	non-squamous	56.7 vs. 30.2	20.0 vs. 9.3	NR vs. 21.1	41 vs. 27	Pembrolizumab/PC vs. carboplatin/pemetrexed	PD-L1 unselected	(Borghaei et al., 2019)
KEYNOTE-189 (NCT02578680)	III	616	non-squamous	47.6 vs. 18.9	9.0 vs. 4.9	22.0 vs. 10.7	71.9 vs. 66.8	Pembrolizumab/PC vs. carboplatin/pemetrexed	PD-L1 unselected	(Gadgeel et al., 2020)
KEYNOTE-407 (NCT02775435)	III	559	Squamous	62.6 vs. 38.4	8.0 vs. 5.1	17.1 vs. 11.6	69.8 vs. 68.2	pembrolizumab/chemotherapy vs. carboplatin/(nab-)paclitaxel	PD-L1 unselected	(Paz-Ares et al., 2019)
IMpower130 (NCT02367781)	III	723	non-squamous	49.2 vs. 31.9	7.0 vs. 5.5	18.6 vs. 13.9	32 vs. 28	Atezolizumab/CnP vs. carboplatin/nab-paclitaxel	PD-L1 unselected	(West et al., 2019)
IMpower131 (NCT02367794)	III	683	Squamous	59.4 vs. 51.3	6.5 vs. 5.6	14.6 vs. 14.3	68 vs. 57	Atezolizumab/CnP vs. carboplatin/nab-paclitaxel	PD-L1 unselected	(Socinski et al., 2018a)
IMpower132 (NCT02657434)	III	578	non-squamous	47 vs. 32	7.6 vs. 5.2	18.1 vs. 13.6	69 vs. 59	Atezolizumab/PC vs. pemetrexed-carboplatin/cisplatin	PD-L1 unselected	(West et al., 2017)
IMpower-150 (NCT02366143)	III	1202	non-squamous	63.5 vs. 48.0	8.3 vs. 6.8	19.2 vs. 14.7	58.5 vs. 50.0	Atezolizumab/BCP vs. bevacizumab/carboplatin/paclitaxel	PD-L1 unselected	(Socinski et al., 2018b)
Combination immune-checkpoint inhibitor and immune-checkpoint inhibitor										
CheckMate-227 (NCT02477826)	III	1739	Squamous and non-squamous	45.3 vs. 26.9	7.2 vs. 5.5	–	31.2 vs. 36.1	Nivolumab/Ipilimumab vs. chemotherapy	PD-L1 unselected	(Hellmann et al., 2018)
						PD-L1≥1% 17.1 vs. 14.9 PD-L1<1% 17.2 vs. 12.2	32.8 vs. 36.1		PD-L1 unselected	(Hellmann et al., 2019)
MYSTIC (NCT02453282)	III	1118	Squamous and non-squamous	–	3.9 vs. 5.4	11.9 vs. 12.9	47.7 vs. 46.0	Durvalumab/Tremelimumab vs. chemotherapy	PD-L1 ⩾ 25%	(Rizvi et al., 2018)

NR, not reached; OS, overall survival; PD-L1, programmed cell-death ligand 1; PFS, progression-free survival; RR, response rate; CnP, carboplatin/nab-paclitaxel; PC, pemetrexed/carboplatin; BCP, bevacizumab plus carboplatin/paclitaxel; ABCP, atezolizumab plus bevacizumab plus carboplatin/paclitaxel; ACP, atezolizumab/carboplatin/paclitaxel.

### Pembrolizumab and Chemotherapy

In the KEYNOTE-021G phase II trial, patients (n = 123) with advanced non-squamous NSCLC not harboring *EGFR* mutations and *ALK* aberrations were recruited. These patients were randomly divided into two arms in 1:1 ratio to receive the following treatments: pembrolizumab combined with carboplatin and pemetrexed and carboplatin and pemetrexed-alone (Langer et al., 2016). The observations indicated the combination therapy to confer better PFS (24.0 months versus 9.3 months) and ORR (56.7 versus 30.2%) than treatment with chemotherapy-alone. Moreover, the combination therapy delayed disease progression and reduced the risk of mortality in the patients (Borghaei et al., 2019). Based on these results, the US FDA, on May 21, 2017, approved the immunotherapy plus chemotherapy regimen, which involves pembrolizumab combined with carboplatin and pemetrexed for the treatment of patients with advanced non-squamous NSCLC not harboring *EGFR*/*ALK* mutations, independent of the expression of PD-L1.

Next, a phase III KEYNOTE-189 trial was conducted to understand the benefit conferred by the administration of pembrolizumab combined with chemotherapy in the patients with advanced non-squamous NSCLC. The patients (n = 616) who were previously-untreated, showed varying expression levels of the PD-L1, and had no *EGFR* mutation or *ALK* rearrangements were included in this trial (Gandhi et al., 2018). They were divided into two arms in a 2:1 ratio to either receive pembrolizumab with pemetrexed and platinum-based chemotherapy or a placebo and pemetrexed and platinum-based chemotherapy. The PFS and OS were set as the primary endpoints for evaluating the outcome of the trial. The latest data presented in the ASCO-2019 meeting suggested that the combinatorial treatment with pembrolizumab plus standard chemotherapy extended the PFS of patients by 4.1 months than the treatment with placebo plus standard chemotherapy. Furthermore, the OS and ORR in the pembrolizumab combinatorial arm were significantly better than that in the placebo arm (OS: 22.0 months versus 10.7 months; ORR: 46.7 versus 18.9%) (Gadgeel et al., 2020). Based on the PD-L1 expression status, the patients were further divided into groups. While the OS was improved to some extent in all the subgroups, those with PD-L1 expression of ≥ 50% showed the maximum clinical benefits. Thus, on the basis of the results of the KEYNOTE-189 clinical trial, the US FDA, on August 20, 2018, approved the combination of pembrolizumab plus pemetrexed-platinum as a first-line treatment of the patients with advanced non-squamous NSCLC (Gandhi et al., 2018).

Further, as opposed to the KEYNOTE-189, the KEYNOTE-407 phase III trial recruited 559 patients with advanced squamous NSCLC who were previously untreated. These patients were randomly divided into two arms to receive chemotherapy, comprised of carboplatin and paclitaxel or nab-paclitaxel, combined with either pembrolizumab or placebo (Paz-Ares et al., 2018). The median follow-up was observed for 7.8 months, and the pembrolizumab combined chemotherapy treatment group was extended by 4.9 months and 1.6 months, respectively, compared to the median OS and median PFS in the placebo combined chemotherapy treatment group (OS: 15.9 months versus 11.3 months; PFS: 6.4 months versus 4.8 months). Based on the outcome of this trial, the US FDA, on October 30, 2018, approved pembrolizumab combined with standard chemotherapy for the treatment of the patients with squamous NSCLC. The final analysis was released in June 2020, which once again demonstrated that the experimental arm of pembrolizumab combined chemotherapy significantly extended OS and PFS (OS: 17.1 months versus 11.6 months; PFS: 8.0 months versus 5.1 months) (Paz-Ares et al., 2020). Moreover, the results of the KEYNOTE-189 were updated at the European Society of Medical Oncology (ESMO) meeting in 2019, where the patients treated with pembrolizumab combined with chemotherapy were shown to have increased ORR and longer PFS and OS (Paz-Ares et al., 2019). Furthermore, the administration of pembrolizumab combinatorial therapy reduced the risk of death in the patients by 29%, indicating its safety. Therefore, in the patients with advanced squamous NSCLC, independent of the PD-L1 expression status, the combination of immunotherapy and chemotherapy may greatly improve the endpoints of the trial and confer a controllable safety.

### Atezolizumab and Chemotherapy

The IMpower series of trials related to the combination treatment of atezolizumab in patients with NSCLC are ongoing. The first phase III clinical trial, IMpower130, which recruited 723 patients with non-squamous NSCLC tumors with *EGFR*/*ALK* wild-type status, was set to evaluate the effectiveness and safety of the combinatorial treatment of atezolizumab than treatment with chemotherapy (carboplatin/nab-paclitaxel)-alone (Cappuzzo et al., 2018). The trial data was updated in 2019. The analysis suggested that the administration of atezolizumab combined with chemotherapy improved the PFS (1.5 months improvement), OS (4.7 months improvement), and ORR (49.2 versus 31.9%) than treatment with chemotherapy-alone (West et al., 2019).

Second, a phase III clinical trial, IMpower131, recruited patients with advanced squamous NSCLC who were previously-untreated. These patients were randomly divided into three arms: Two of the experimental arms received atezolizumab combined with chemotherapy (carboplatin/nab-paclitaxel), while the third control arm received chemotherapy-alone. The results suggested that patients in the two experimental arms showed prolonged PFS than in those in the control arm. The PFS was significantly higher in patients with high expression of PD-L1 than in other patients. However, no significant difference was observed in OS of patients in the experimental arms (Socinski et al., 2018a).

Third, the IMpower132 phase III trial recruited the patients with advanced-stage non-squamous NSCLC harboring no *EGFR*/*ALK* mutations. These patients received either the atezolizumab combined with chemotherapy (pemetrexed plus carboplatin/cisplatin) or chemotherapy-alone (West et al., 2017). The recent analysis suggested that the combinatorial treatment arm showed longer PFS, but no significant improvement in the OS, than in the chemotherapy-alone arm (Papadimitrakopoulou et al., 2018).

In the IMpower150 phase III trial, patients (n = 1202) with non-squamous NSCLC were recruited independent of the expression levels of the PD-L1. The patients were divided into three arms based on the proportion and received different treatments: the first arm received atezolizumab combined with chemotherapy (CP: carboplatin/paclitaxel), the second arm received atezolizumab plus anti-angiogenesis drug (bevacizumab) combined with chemotherapy (ABCP), and the third arm received anti-angiogenesis drugs and chemotherapy (BCP). The results suggested that the administration of the ABCP could effectively prolong the PFS (1.5 months) and OS (4.5 months) than with BCP (Socinski et al., 2018b). The trial suggested that the administration of the ABCP quadruple therapy could prolong the survival duration of the non-squamous NSCLC patients. Therefore, the US FDA, on December 6, 2018, approved the ABCP as a first-line immunotherapy in patients with non-squamous NSCLC not harboring *EGFR*/*ALK* mutations, independent of the expression levels of the PD-L1. Furthermore, the subgroups with sensitized *EGFR*/*ALK* mutations were analyzed, which led to the conclusion that the use of ABCP quadruple therapy failed to confer significant PFS (9.7 months versus 6.1 months), and the median OS could not be achieved (NR versus 17.5 months). However, in the intention-to-treat (ITT) subgroup, the OS was found to be prolonged by 5 months (19.8 months versus 14.9 months) (Reck et al., 2019c). Thus, based on the existing experimental data, the EMA has approved the administration of atezolizumab combined with anti-angiogenesis drugs plus chemotherapy for the treatment of non-squamous NSCLC patients harboring the *EGFR*/*ALK* mutations who failed to respond to the first-line molecular targeted therapy.

## First-Line PD-1/PD-L1 Checkpoint Inhibitors Combined With CTLA4 Checkpoint Inhibitors for NSCLC

The previous clinical trials have shown that targeting checkpoint pathway have anti-tumor effects. However, the experimental studies addressing the combined inhibition of these pathways to the tumorigenic activity are ongoing. The **Table 3** summarizes the observations of the ongoing phase III clinical trials, *viz.* the CheckMate-227 and MYSTIC.

### Nivolumab and Ipilimumab

In the phase III CheckMate-227 trial, patients (n = 1739) with advanced NSCLC not harboring the *EGFR* mutations and *ALK* rearrangements, and who were previously-untreated, were recruited in 2018. These patients were divided into two experimental arms based on the expression levels of the PD-L1, as the PD-L1 positive and PD-L1 negative arm. Further, each experimental arm was divided into three subgroups at a ratio of 1:1:1 and they received different treatments, respectively. In the experimental arm with the PD-L1 positive expression, patients received nivolumab plus ipilimumab combinatorial immunotherapy, nivolumab monotherapy, and chemotherapy-alone, respectively. Whereas, patients in the PD-L1 negative arm received dual immunotherapy, nivolumab combined with chemotherapy, and chemotherapy-alone (Hellmann et al., 2018). To compare the efficacy of nivolumab plus ipilimumab dual immunotherapy versus chemotherapy-alone, the PFS and OS were selected as two main endpoints for the diverse population. Further, PFS was used to evaluate the patients with high TMB, while OS was used to assess those with positive PD-L1 expression. The results suggested that irrespective of the PD-L1 expression, patients with high TMB upon treatment with dual immunotherapy showed significantly better PFS and increased ORR than upon treatment with chemotherapy-alone (PFS: 7.2 months versus 5.5 months; ORR: 45.3 versus 26.9%) (Hellmann et al., 2018). Therefore, these results support the use of TMB as a biomarker to predict the efficacy of treatment for the patients with NSCLC. Furthermore, the updated results in 2019 showed that in patients with positive PD-L1 expression, the OS upon combined use of nivolumab plus ipilimumab dual immunotherapy was 2.2 months longer than that in the chemotherapy-alone arm (17.1 months versus 14.9 months). Moreover, in patients with negative PD-L1 expression, the OS benefit was more pronounced when treated with dual immunotherapy (17.2 months versus 12.2 months). Taken together, independent of the TMB and expression of PD-L1, the administration of nivolumab plus ipilimumab dual immunotherapy conferred different degrees of clinical benefit (17.1 months versus 13.9 months) in the patients. Additionally, the median duration of response was found to be significantly better in the patients treated with nivolumab plus ipilimumab dual immunotherapy than when treated with chemotherapy-alone (23.2 months versus 6.2 months) (Hellmann et al., 2019). Therefore, these results support and validate the “chemotherapy-free” first-line treatment regimen for the patients with advanced NSCLC.

### Durvalumab and Tremelimumab

Further, the phase III MYSTIC clinical trial evaluated the safety and effectiveness of the treatment regimens related to durvalumab. The patients (n = 1118) were recruited and divided into three arms in equal proportions to receive durvalumab monotherapy, durvalumab plus tremelimumab dual immunotherapy, and chemotherapy-alone. Here, patients with PD-L1 expression > 25% were considered, and the OS and PFS were considered as the main endpoints for evaluating the efficacy of durvalumab single-therapy versus chemotherapy-alone and dual immunotherapy versus chemotherapy-alone, respectively (Rizvi et al., 2018). The results suggested that the OS and PFS were statistically insignificant for all the comparisons in the patients with advanced and metastatic NSCLC. However, the OS in patients treated with first-line durvalumab immunotherapy was better, and more clinical trials would be required to ascertain its efficacy in NSCLC.

## Biomarkers Predictive of Efficacy to First-Line Treatment

As mentioned in the introduction, majority of the patients are insensitive to immunotherapy and fail to show survival benefits. Therefore, it is imperative to perform immune monitoring of the clinical trials to identify biomarkers that can distinguish between potential responders and non-responders. According to the screened groups of potential beneficiaries, the use of immunotherapy can maximize the therapeutic effect. In this section, the recent predictive biomarkers, including the PD-L1 and TMB have been studied.

### PD-L1 Expression

The PD-L1 has been considered as one the most common predictive biomarkers in NSCLC immunotherapy. Multiple clinical diagnosis and treatment guidelines recommend the use of immunohistochemistry (IHC) methods to detect the expression level of PD-L1, which can be used to screen potential benefit populations and predict efficacy. At present, a range of PD-L1 detection commercial kits for different epitopes have been developed, including 22C3, 28-8, SP142, SP263 and 73-10. The above five antibodies are detected on two immunohistochemistry platforms Dako and Ventana respectively, and the evaluation cell types include tumor cells (TC) and/or tumor-infiltrating immune cells (ICs). Four antibodies (22C3, 28-8, SP263, and SP142) have been approved by the US FDA, and each immune checkpoint inhibitors uses a different antibody to evaluate PD-L1 expression levels. For instances, pembrolizumab uses 22C3 clone antibody and atezolizumab uses SP142 clone antibody as companion diagnosis, nivolumab uses 28-8 clone antibody, and durvalumab uses SP263 clone antibody as complementary diagnosis (Büttner et al., 2017). Moreover, for NSCLC clinical trials at various stages, different detection antibodies or platforms and different immune checkpoint inhibitors adopt various cut-off values ​​and scoring systems to define the expression level of PD-L1 (Lantuejoul et al., 2020; Tumor Pathology Committee of Chinese Anti-Cancer Association et al., 2020). In **Table 4**, we summarize the IHC PD-L1 assay methods for NSCLC patients.

**Table 4:** Summary of IHC PD-L1 assay in patients with NSCLC.

PD-L1 detection antibody	Type of antibody	Diagnostic platform	Evaluation of cell types	PD-L1 Cut-off	Immunotherapy drug	FDA approved
22C3	Mouse monoclonal antibody	Dako Link 48	TC	⩾ 1%, ⩾ 50%	Pembrolizumab	Companion diagnostic
28-8	Rabbit monoclonal antibody	Dako Link 48	TC	⩾ 1%, ⩾ 5%	Nivolumab	Complementary diagnostic
SP142	Rabbit monoclonal antibody	Ventana Benchmark or Ultra	TC and/or IC	TC: ⩾ 1%, ⩾ 5%, ⩾ 50% IC: ⩾ 1%, ⩾ 5%, ⩾ 10%	Atezolizumab	Companion diagnostic
SP263	Rabbit monoclonal antibody	Ventana Benchmark or Ultra	TC	⩾ 25%	Durvalumab	Complementary diagnostic
73-10	Rabbit monoclonal antibody	Dako Link 48	TC	⩾ 1%, ⩾ 5%, ⩾ 80%	Avelumab	Diagnostic test

FDA, U.S. Food and Drug Administration; PD-L1 programmed cell death ligand 1; TC, Tumor cells; IC, Infiltrating immune cells.

In the phase IB KEYNOTE-001 clinical trial, patients with PD-L1 ≥ 50% showed significant clinical and survival benefits upon administration of pembrolizumab single drug treatment. Moreover, this trial proved that the expression of the PD-L1 may indicate the degree of clinical benefit in the patients (Garon et al., 2015). Further, the analysis of the phase III KEYNOTE-024 and KEYNOTE-042 clinical trials suggested that in the patients with positive PD-L1 expression, especially in those with PD-L1 expression ≥ 50%, the pembrolizumab monotherapy significantly increased the OS of patients than in the chemotherapy-alone arm. Moreover, along with better OS, patients showed lower incidence of the TRAEs (Reck et al., 2016; Mok et al., 2019a). Furthermore, the analysis of multiple clinical trials related to the PD-1/PD-L1 checkpoint inhibitors indicated that when the expression levels of PD-L1 were different, the survival benefits in NSCLC patients were variable. This indicated that the patients with high expression of PD-L1 may show better survival benefits and longer survival duration than those with lower expression. Therefore, the expression of PD-L1 may serve as a biomarker to predict the degree of benefit of the PD-1/PD-L1 inhibitors in different patients. However, there has been a lack of uniformity in the kits used to determine the PD-L1 expression across institutions and departments (Büttner et al., 2017). Thus, studies have even tested the consistency between different detection methods. For instance, the clinical trials that used the 28-8, 22C3 and SP263 kits showed consistency and reproducibility, while the SP142 kit showed inaccuracy in predicting the PD-L1 expression in the tumor cells (Adam et al., 2018). Additionally, the use of the PD-L1 expression as a qualitative variable would explain the variable thresholds obtained for stratification of the patients, and hence, the varying immunotherapy strategies adopted in the clinical trials. Although determining the PD-L1 expression has become a routine test, the temporal and spatial heterogeneity in the expression of PD-L1 and several other challenges affect its efficacy as a predictive biological marker in the NSCLC patients.

In addition, the research results published by R de Vries et al. in October 2019 showed that the molecular characteristics of exhaled air may capture the inflammatory environment related to the individual’s response to the PD-1 treatment, thereby screening out patients with NSCLC that can produce sustained immunotherapy responses. On the other hand, this study may prevent ineffective treatment among those who have been identified as non-responders to immunotherapy (de Vries et al., 2019). We hope that electronic nose assessment will become a widely used predictive biomarker soon.

### Tumor Mutation Burden

The cancer develops by the gradual accumulation of numerous somatic mutations in the body (Gubin et al., 2015). While the incidence of mutation varies across tumor types, the NSCLC are considered the most mutated malignant tumors (Branca, 2016). When a mistranslation mutation occurs, the protein will be translated abnormally and expressed abnormally, and tumors with high tumor mutation burden (TMB) will be recognized as a new antigen by the immune system (Braun et al., 2016). The administration of immune-checkpoint inhibitors can aid the immune system clear the tumor cells. The TMB can be defined as the number of mutations per megabase (Mb) of the DNA, which is determined using the DNA sequencing. The whole-genome sequencing (WGS) and whole-exome sequencing (WES) are the sequencing methods used to determine the TMB (Greillier et al., 2018). The TMB has been used as a predictive biomarker to evaluate the role of immunotherapy, mainly nivolumab, in clinical trials. The phase I CheckMate-012 clinical trial studied the combinatorial efficacy of ipilimumab and nivolumab in patients with advanced NSCLC. The results suggested that the dual ipilimumab and nivolumab immunotherapy conferred longer PFS and better ORR in patients with high TMB (TMB ≥ 10 mut/Mb) than in patients with low TMB (TMB < 10 mut/Mb) (Hellmann et al., 2018). Further, the phase II CheckMate-568 clinical trial suggested that independent of the expression status of the PD-L1, the patients showed an increase in the ORR with the gradual increase in the TMB. When TMB expression reaches 10 or higher, the ORR entered a platform period is not increasing. Moreover, the TMB is above a certain value (TMB ≥ 10mut/Mb) corresponds to longer PFS in the patients (Ready et al., 2019). The analysis of several phase-III clinical trials, such as the CheckMate-227 and CheckMate-026, suggested that the TMB could potentially predict the efficacy of the immunotherapy in the patients with NSCLC. However, several issues impede the utility of TMB, including the long test cycles and high-cost and standardization of the threshold for high- and low-TMB.

## Conclusion

**Figure 3:**
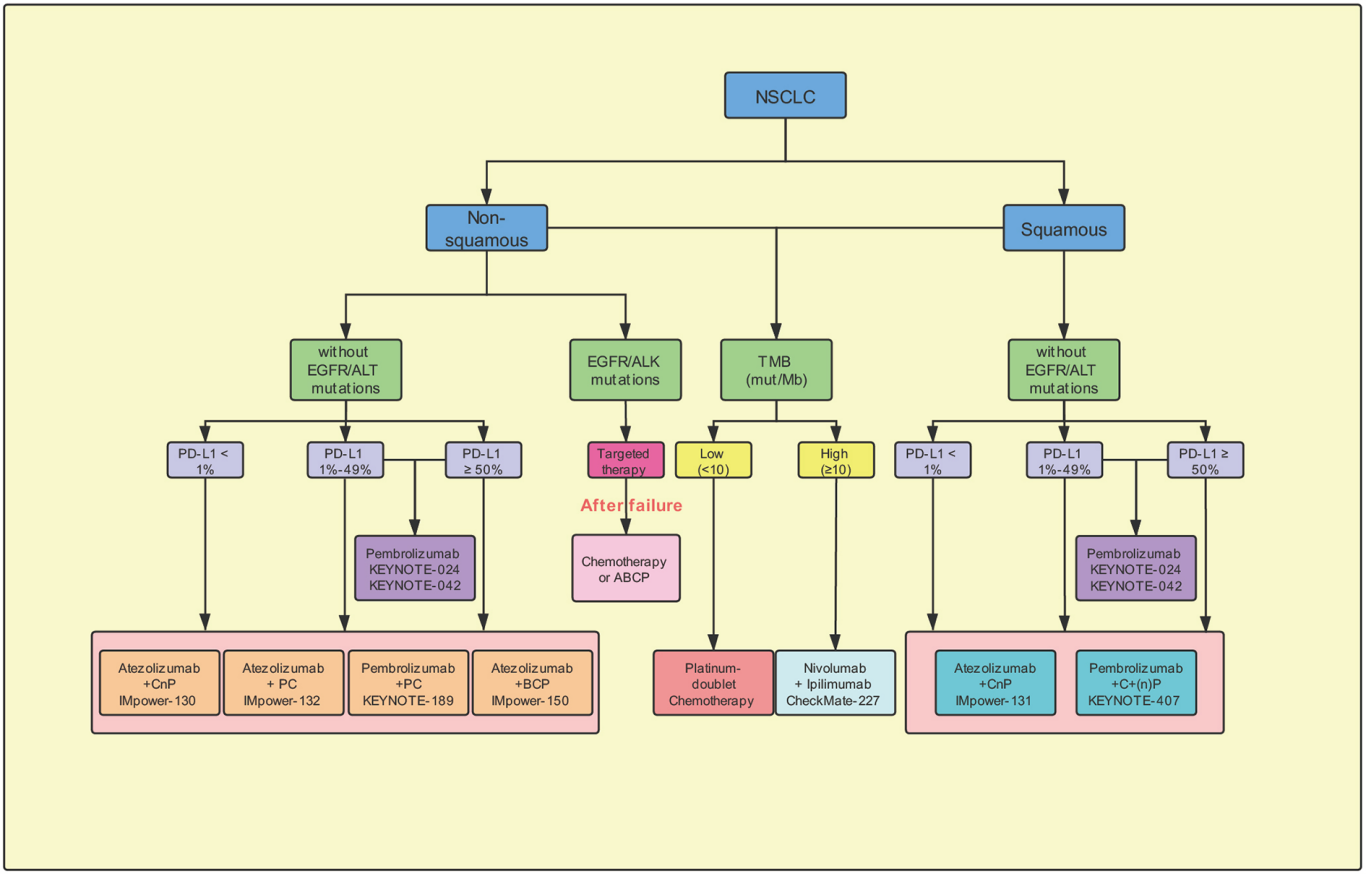
Potential suggestion for first-line immunotherapy options for advanced non-small-cell lung cancer. CnP, carboplatin/nab-paclitaxel; PC, pemetrexed/carboplatin; BCP, Bevacizumab plus carboplatin/paclitaxel; ABCP, Atezolizumab plus bevacizumab plus carboplatin/paclitaxel; ACP, Atezolizumab/carboplatin/paclitaxel; EGFR, epidermal growth factor receptor; ALK, anaplastic lymphoma kinase; Mb, megabase; mut, mutations; PD-L1, programmed cell-death ligand 1; TMB, tumor mutation burden.

The immunotherapy has the potential to modify the treatment regimen and outcome in the patients with NSCLC. Based on the results of the phase III CheckMate-017 and CheckMate-057 clinical trials, the US FDA, in 2015, approved nivolumab as a second-line treatment post chemotherapy in the patients with advanced squamous and non-squamous NSCLC (Brahmer et al., 2015; Borghaei et al., 2015). The results suggested that the administration of nivolumab monotherapy effectively increased the ORR and conferred significantly better OS than treatment with second-line docetaxel. In this review, we have described the details of the completed and ongoing clinical trials, which should aid in exploring the appropriate first-line, single-drug or combinatorial, treatment in the previously-untreated patients with advanced NSCLC not harboring *EGFR*/*ALK* mutations (**Figure 3**). In NSCLC, different first-line immunotherapy strategies have been selected depending on the expression of the PD-L1. The clinical studies recommend the administration of pembrolizumab as a monotherapy or combined with chemotherapy based on the expression levels of PD-L1 ≥ 50% in the NSCLC patients. Furthermore, in the patients with PD-L1 < 50%, results support the administration of pembrolizumab combined with chemotherapy. Whereas, pembrolizumab-alone is being administered in those unwilling to or unsuitable for receiving chemotherapy. Thus, in the future, as additional clinical trials attain the set primary endpoint and obtain adequate data support, the choice of first-line immunotherapy would become more diverse.

The results of the ASCO and WCLC published in 2019 indicated a 10% improvement in the 5-year OS of the patients treated with immunotherapy. However, several problems exist in the immunotherapeutic treatment. First, an increasing number of clinical trials have been initiated to elucidate the role of immunotherapy in the patients belonging to stage IB–IIIB of NSCLC. At the 2019 ASCO meeting, three neoadjuvant immunotherapy studies were announced, including the LCMC3, NEOSTAR, and NADIM (Cascone et al., 2019; Kwiatkowski et al., 2019; Provencio et al., 2019). The clinical trial LCMC3 was mainly initiated to evaluate the patients with NSCLC after neoadjuvant treatment with atezolizumab. We included patients with stages IB to selected IIIB resectable NSCLC into experimental studies to the safety and efficacy of neoadjuvant therapy. The interim data showed that the major pathological response (MPR) rate and the pathological complete response (pCR) rate to be 19 and 5%, respectively. Next, the neoadjuvant therapy NEOSTAR clinical trial with nivolumab and ipilimumab confirmed that in patients with surgically resectable NSCLC, the survival benefits conferred by the combinatorial neoadjuvant therapy were significantly better than those upon treatment with nivolumab monotherapy. Further, the neoadjuvant therapy NADIM clinical trial mainly recruited the patients with stage IIIA NSCLC, and for the first time explored the neoadjuvant treatment plan combining immunotherapy and chemotherapy. The results indicated that the administration of neoadjuvant immunotherapy decreased the stage in most of the patients, and the MPR and pCR rates were 85.36 and 71.4%, respectively. Although the inclusion of neoadjuvant immunotherapy, especially combined with chemotherapy, has improved the pCR, a series of problems impede its utility, such as the criteria for efficacy evaluation, choice of surgical timing, and postoperative treatment options.

Second, in order to screen out potential populations who may benefit from immunotherapy, exploring biomarkers that can effectively predict the efficacy of immunotherapy is one of the key steps. At present, the main biomarkers used in clinical practice of NSCLC immunotherapy include PD-L1 expression and tumor mutation burden (TMB). Compared with the detection of TMB, the detection of PD-L1 expression level has the advantages of simplicity, convenience and low price, so that it is more widely used in practical work. Another relatively common important marker is microsatellite instability (MSI) (Lemery et al., 2017). MSI needs to detect the expression of 4 mismatch repair proteins, while polymerase chain reaction can detect 5 microsatellite loci. Previous studies have shown that tumors with high microsatellite instability (MSI-H) also have a higher tumor mutation burden, indicating that there is a certain correlation between them. Moreover, a series of experimental studies have confirmed that tumor infiltrating lymphocytes (TILs), neutrophil-to-lymphocyte ratio (NLR), electronic nose analysis of exhaled breath and other biomarkers can help to screen patients who can benefit from immunotherapy for NSCLC (Tokito et al., 2016; de Vries et al., 2019; Lee and Ruppin, 2019; Rossi et al., 2020).

In addition, the criteria for evaluating the immunotherapy are not well-defined. The Response Evaluation Criteria in Solid Tumors 1.1 (RECIST1.1), which is currently being used for the evaluation of the efficacy of the treatment, is primarily based on the change in tumor size in the imaging. However, it may underestimate the benefit of immunotherapy in the patients, and hence affect the evaluation. Therefore, the international RECIST working group has formally proposed newer standards for evaluating the efficacy of the treatment, such as the Immune-related RECIST (irRECIST) and immune-related pathologic response criteria (irPRC) (Cottrell et al., 2018; Tazdait et al., 2018). Since the evaluation criteria for the immunotherapy would get constantly updated, the accuracy and effectiveness of the evaluation criteria remain to be completely verified.

Finally, the occurrence and management of IRAEs needs to be thoroughly evaluated. Though the administration of checkpoint inhibitors can prolong the survival duration in patients, it can also change their immune homeostasis. The disruption of the immune homeostasis would result in a series of autoimmune side effects, termed as the IRAEs (Puzanov et al., 2017). Moreover, while the overall incidence of IRAEs is low, few of them can have consequences and, thus, require attention and active prevention.

The conclusions of several clinical trials administering checkpoint inhibitors for treatment of cancer indicate IRAEs related to the endocrine toxicity (thyroid dysfunctions, hypophysitis, adrenal insufficiency, and pituitary), gastrointestinal tract (diarrhea and colitis), lungs (pneumonia), skin (rash and pruritus), and joints (arthritis). In the NSCLC, the incidence of pulmonary IRAEs is higher; for example, the incidence of pneumonitis ranks first, which may be related to chronic obstructive airway disease or previous treatment with chemotherapy in the NSCLC patients (Khoja et al., 2017). In 2019, the Chinese Society of Clinical Oncology (CSCO) published the guidelines for the toxicity management of the immune-checkpoint inhibitors that deal with basic management principles of the IRAEs, including the prevention, detection, evaluation, treatment, and monitoring (Zhou et al., 2020). Thus, the early identification would detect and manage adverse events, and prevent the fatal outcomes in some cases.

In summary, the field of immunotherapy has shown rapid development since the US FDA approval in 2015 to administer the immunotherapeutic drugs as second-line, and recently as first-line, treatment in the patients with advanced stage NSCLC. However, the strategies are in their early phases and continue to suffer serious challenges. Therefore, we can anticipate that a systematic treatment model based on immunotherapy, along with the multidisciplinary approach, inclusive of surgery, radiotherapy, and supportive treatment would ensure the selection of the most appropriate treatment for the patients with different stages of the NSCLC.

## Author Contributions

ZH and WS conceived and designed the study. ZH wrote the paper. ZH, TL, YD, and YQ jointly designed the figures. DL and YW jointly designed the tables. ZH, YW, LS, and WJ reviewed and edited the manuscript. All authors contributed to the article and approved the submitted version.

## Funding

This research was funded by Key R & D programs in Shandong Province (grant number: 2018GSF118119).

## Conflict of Interest

The authors declare that the research was conducted in the absence of any commercial or financial relationships that could be construed as a potential conflict of interest.
